# Cannabidiol (CBD) Inhibited Rhodamine-123 Efflux in Cultured Vascular Endothelial Cells and Astrocytes Under Hypoxic Conditions

**DOI:** 10.3389/fnbeh.2020.00032

**Published:** 2020-03-17

**Authors:** Jerónimo Auzmendi, Pablo Palestro, Agustín Blachman, Luciana Gavernet, Amalia Merelli, Alan Talevi, Graciela Cristina Calabrese, Alberto Javier Ramos, Alberto Lazarowski

**Affiliations:** ^1^Instituto de Fisiopatología y Bioquímica Clínica, Facultad de Farmacia y Bioquímica, Universidad de Buenos Aires, Buenos Aires, Argentina; ^2^Consejo Nacional de Investigaciones Científicas y Técnicas, Buenos Aires, Argentina; ^3^Laboratorio de Investigaciones Bioactivas y Desarrollo, Departamento de Ciencias Biológicas, Facultad de Ciencias Exactas, Universidad de La Plata, La Plata, Argentina; ^4^Cátedra de Biología Celular y Molecular, Departamento de Ciencias Biológicas, Facultad de Farmacia y Bioquímica, Universidad de Buenos Aires, Buenos Aires, Argentina; ^5^Laboratorio de Neuropatología Molecular, Instituto de Biología Celular y Neurociencia “Prof. E. De Robertis,” Facultad de Medicina, Universidad de Buenos Aires, Buenos Aires, Argentina

**Keywords:** cannabidiol, P-glycoprotein, hypoxia, endothelial cells, astrocytes

## Abstract

Despite the constant development of new antiepileptic drugs (AEDs), more than 30% of patients develop refractory epilepsy (RE) characterized by a multidrug-resistant (MDR) phenotype. The “transporters hypothesis” indicates that the mechanism of this MDR phenotype is the overexpression of ABC transporters such as P-glycoprotein (P-gp) in the neurovascular unit cells, limiting access of the AEDs to the brain. Recent clinical trials and basic studies have shown encouraging results for the use of cannabinoids in RE, although its mechanisms of action are still not fully understood. Here, we have employed astrocytes and vascular endothelial cell cultures subjected to hypoxia, to test the effect of cannabidiol (CBD) on the P-gp-dependent Rhodamine-123 (Rho-123) efflux. Results show that during hypoxia, intracellular Rho-123 accumulation after CBD treatment is similar to that induced by the P-gp inhibitor Tariquidar (Tq). Noteworthy, this inhibition is like that registered in non-hypoxia conditions. Additionally, docking studies predicted that CBD could behave as a P-gp substrate by the interaction with several residues in the α-helix of the P-gp transmembrane domain. Overall, these findings suggest a direct effect of CBD on the Rho-123 P-gp-dependent efflux activity, which might explain why the CBD add-on treatment regimen in RE patients results in a significant reduction in seizure frequency.

## Introduction

Refractory epilepsies (REs) are characterized by high recurrence of seizures that cannot be controlled by at least two well-tolerated antiepileptic drugs (AEDs) appropriate for the particular epilepsy type (Kwan and Brodie, 2010). Whereas the continuous development of new AEDs has offered important improvements regarding treatment adherence, pharmacokinetics, tolerability, and efficacy in certain particular epilepsy types (Löscher et al., 2013; Hanaya and Arita, 2016; Talevi, 2016), similar percentage of non-responder patients remains the same as that observed for the AEDs since two centuries ago (Kwan and Brodie, 2000). Several hypotheses to explain this common multidrug-resistant (MDR) phenotype in RE have been proposed. Among them, the “transporter hypothesis” and the “pharmacokinetics hypothesis” suggest that ABC transporters could play a central role in RE (Leandro et al., 2019). Both hypotheses could explain the first pioneering description of a MDR phenotype in an RE with persistent low levels of AEDs in plasma associated with P-glycoprotein (P-gp) overexpression (Tishler et al., 1995; Lazarowski et al., 1999, 2004a). P-gp, so far the most studied of these transporters, is encoded by ABCB1 gene. P-gp expression is regulated by hypoxia, inflammation, and stress-related transcriptional factors such as HIF-1α, NFκB, STAT3, or PXR (Ho and Piquette-Miller, 2006; Jain et al., 2014; Zhang Z.-L. et al., 2018). Moreover, upregulation of P-gp was reported in a wide spectrum of experimental models and clinical studies of RE (Lazarowski et al., 2004b, 2007b; Hartz et al., 2017; Deng et al., 2018; Weidner et al., 2018). During hypoxia, P-gp was overexpressed in different tissues, including brain and heart (Lazarowski et al., 2007a; Aviles-Reyes et al., 2010; Merelli et al., 2011a, b). Remarkably, this overexpression was also registered in brain and heart after repetitive seizures and status epilepticus, showing a hypoxic–ischemic scenario (Auzmendi et al., 2014, 2018).

Some alternative therapies are eligible, before the highly invasive surgical removal of the epileptic focus, to improve life quality of patients with RE. Vagal nerve stimulation or ketogenic diet has been applied with variable outcomes (Johnson and Wilson, 2018; Liu et al., 2018; D’Andrea Meira et al., 2019; Dibué-Adjei et al., 2019; Hwang et al., 2019). More recently, the use of different Cannabis compounds to treat seizures has emerged as a potential therapeutic opportunity. Particularly, Cannabis compounds are employed to control the so-called catastrophic epilepsies in children (Maa and Figi, 2014). Several reports and reviews have highlighted the benefits of a rational and selective use of these compounds for the treatment of RE (Filloux, 2015; Rosenberg et al., 2015; Friedman and Devinsky, 2016; Chen et al., 2018). Among the wide spectrum of Cannabis compounds, cannabidiol (CBD) has been suggested as a relevant candidate for RE control because of its no psychoactive effect unlike those produced by Δ^9^-tetrahydrocannabinol (Tasker et al., 2015). Recently, the Food and Drug Administration (FDA) and the European Medicines Agency (EMA) have recently approved oral CBD as an add-on treatment for Dravet and Lennox–Gastaut syndromes (Devinsky et al., 2019).

Cannabis compounds act through specific endocannabinoid receptors (CB1, CB2) (du Plessis et al., 2015; Basavarajappa et al., 2017). In the central nervous system (CNS), the CB1 receptor is widely expressed in neurons of hippocampus and neocortex, while glial cells express both CB1 and CB2 (Scotter et al., 2010; Maccarrone et al., 2011). Interestingly, the stimulation of the endocannabinoid system plays an important role in a variety of physiological functions and conditions such as neuronal development and plasticity, food intake, energy balance, and cell apoptosis (Marzo et al., 2004; Moreno et al., 2005; Pacher et al., 2006). More recently, it has also been proposed that CBD action involves non-canonical targets such as the transient receptor potential (TRP) channel (Hassan et al., 2014; Iannotti et al., 2014), the adenosine receptor A2A (A2AAR) (Ohta and Sitkovsky, 2001), and the orphan receptor (GPR55) (Chiurchiù et al., 2015) in addition to its known activity on CB1. Furthermore, CBD has also been reported to elicit anti-inflammatory effects (Booz, 2011; Burstein, 2015) that may decrease the glial response after seizures. Besides, CBD activates the microglia promoting their phagocytic activity (Hassan et al., 2014). To date, few studies have assayed CBD and other Cannabis compounds as blockers, substrates, or modulators of the expression of P-gp and other ABC transporters (Holland et al., 2006, 2009; Zhu, 2006; Feinshtein et al., 2013; Brzozowska et al., 2016). Despite the fact that CB1 activation by seizure-induced release of endocannabinoids could play a neuroprotective role (Alger, 2004), its effects on the MDR phenotype in RE remain to be disclosed. Inhibition of P-gp and other ABC transporters could facilitate the normal action of AEDs, bringing new opportunities to improve seizure control with classical AEDs in patients with RE (Höcht et al., 2007; Feinshtein et al., 2013; Hisham et al., 2018; Xie et al., 2018; Zhang H.-L. et al., 2018). Consequently, these transporters emerge as potential new pharmacological targets in RE (Robey et al., 2008).

The aim of this study was to evaluate the possible inhibitory effect of CBD on the active efflux of the fluorescent P-gp-substrate Rhodamine-123 (Rho-123) by *in vitro* studies. Taking into account all above described, P-gp overexpression at the blood–brain barrier (BBB) level can limit the access of AEDs to the brain parenchyma. Cultures of both members of neurovascular unit (NVU) astrocytes and vascular endothelial cells were analyzed in hypoxia condition. CBD inhibitory effect on P-gp activity was tested through Rho-123 efflux assay, comparing it with that of the highly specific P-gp inhibitor Tariquidar (Tq). Additionally, *in silico* studies were performed to explore and predict a possible direct interaction between P-gp and CBD as a substrate/competitive inhibitor.

## Materials and Methods

### Ethics Statement

All procedures involving animals and their care were conducted in accordance with our institutional guidelines, which comply with the NIH guidelines for the Care and Use of Laboratory Animals and the principles presented in the Guidelines for the Use of Animals in Neuroscience Research by the Society for Neuroscience, and were approved by the CICUAL committee (0092357/2019) of the School of Medicine of the University of Buenos Aires. All efforts were made to minimize animal suffering and to reduce the number of animals used.

### Reagents

Cell culture reagents were obtained from Invitrogen Life Technologies (Carlsbad, CA, United States). Fetal calf serum (FCS) was purchased from Natocor (Córdoba, Argentina). Poly-L-lysine, Rhodamine 123, third-generation P-gp inhibitor Tq, and other chemicals were obtained from Sigma–Aldrich (United States). CBD was obtained from Enecta.

### Glial Culture

Primary cortical glial cell cultures were obtained from nine 3–5 postnatal day Wistar rats as described previously (Merelli et al., 2019). Briefly, rats were decapitated, and brains were surgically removed from the skull under sterile condition. Using a fine tip tweezer, meninges were eliminated, and brain cortices were removed and mechanically disrupted within Dulbecco’s modified Eagle medium (DMEM). After several centrifugations, dissociated glial cells were resuspended in DMEM supplemented with 10% FCS, 2 mM L-glutamine, and 100 μg/ml penicillin–streptomycin. Then, 1.5 × 10^4^ cells/ml glial cells were plated in 96-multi-well plate, incubated in supplemented DMEM at 37°C, 5% CO_2_. The medium was exchanged every 48 h until the cells reached 70–80% confluence.

### Vascular Endothelial Cell Culture

Cells derived from polyoma middle T-transformed murine heart endothelium (H5V) (Calabrese et al., 2011) were grown in DMEM supplemented with 10% FBS, streptomycin (100 mg/ml), and penicillin (100 UI/ml) in 5% CO_2_ atmosphere, at 37°C. The medium was exchanged every 48 h until the cells reached 70–80% confluence.

### Chemical Hypoxia Induction

**FIGURE 1:**
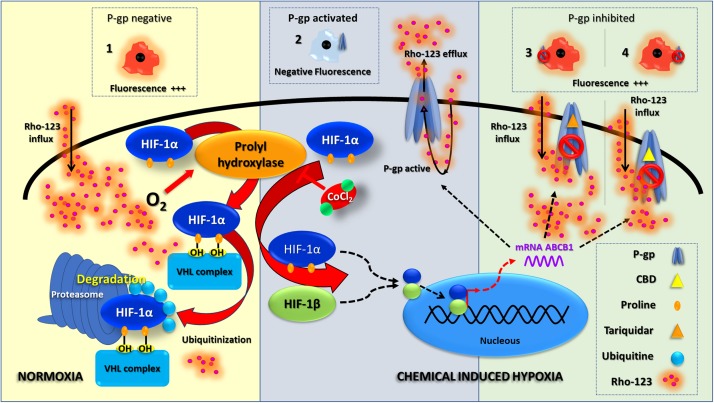
Chemical hypoxia induction. This scheme summarizes the HIF-1 molecular pathway under normoxia and chemical-induced hypoxia; this also shows an interpretation of Rho-123 efflux assay results. Under normoxia, P-gp is absent and a negative result of Rho-123 efflux assay is shown as an increased fluorescence (1). Since the chemical hypoxia is induced, a stabilization of HIF-1 leads to a P-gp overexpression; thus, a positive result of Rho-123 efflux assay is related to a decreased fluorescence (2). The inhibition of P-gp activity (with TQ or CBD—3 and 4, respectively) correlates with an increase in fluorescence.

Glial and endothelial cells (1.5 × 10^6^ cells/ml) were depleted of serum for 4 h before incubating with or without 0.3 mM CoCl_2_ in medium supplemented with 0.5% FBS for 6 h (final volume 5 ml). Figure 1 summarizes the experimental scheme and the molecular mechanisms of chemical hypoxia induction.

### Rho-123 Efflux Assay

This approach to test the P-gp activity was performed as described previously (Merelli et al., 2019). Briefly, following the chemical hypoxia induction, glial and endothelial cells were incubated in the presence of increasing concentration CBD for 30 min [5, 50, or 100 μM, prepared from a stock solution of 0.1 mM in dimethyl sulfoxide (DMSO)].Then, Rho-123 (1 μM in DMEM) was added for 5 min. Controls were carried out in the absence of CBD or with Tq (5 μM) used as a specific blocker of P-gp. All treatments were performed in triplicate. Figure 1 summarizes the experimental scheme and the molecular mechanisms of Rho-123 efflux studies.

### Computational Studies

In previous investigations, a human P-gp homology model was constructed based on the structure encoded as 3G61 in Protein Data Bank, which showed the highest sequence coverage (92%) and percent of identity (82%) among other alternatives. Comparison of several docking software and conditions (see next paragraph) allowed us to conclude that Autodock Vina flexible docking was the best choice among the tested options. As further validation, the model was subsequently used to find new anticonvulsant compounds that do not interact with P-gp and might then constitute potential therapeutic options to treat refractory patients with high expression levels of such transporter (Jäger et al., 1997; Semenza, 2009; Parasrampuria and Mehvar, 2010).

To select the best docking protocol, we run different simulations (by changing the docking software and/or conditions) to predict the interactions with P-gp of a dataset of compounds. The dataset comprised small molecules with known interaction with the transporter (from now on, binders) and structures with no interaction with the protein (non-binders). Using the docking score as discriminating variable, we analyzed the capacity of different docking protocols to classify them correctly; that is, the non-binders should have higher docking score than binders. We quantified this information through the area under the receiver operating characteristic (ROC) curves (Palestro et al., 2018). This metric also allowed us to decide the threshold score that can be used to discern between the classes. Three docking software were tested: Glide (version 5.7, Schrodinger Suite 2011), Autodock4.2, and Autodock Vina (The Scripps Research Institute^1^) (Trott and Olson, 2010). The “docking active site” was defined through a 24 × 24 × 24 Å^3^ grid, centered on the relative position of the ligand in the crystallographic structure of mouse P-gp (PDB code 3G61). Such region comprises the entire transmembrane region, since the binding subsites for P-gp substrates and inhibitors reside in this area (Aller et al., 2009).

In terms of flexibility of the target, we run both rigid and flexible docking simulations. The flexibility of the target was considered by allowing two different sets of amino acids to move in the simulations. In one system, we set the binding site residues Phe-335, Phe-343, Phe-728, Phe-732, and Phe-978 as flexible (model A), whereas in the other simulation, we selected as flexible Tyr-307, Tyr-953, Phe-343, and Phe-978 (model B). The selection of the mobile residues in model A was founded on the analysis of the amino acids that interact with the ligands in the experimental mouse complexes (PDB codes 3G60 and 3G61). For model B, we analyzed first the conformation of the flexible residues in model A after the docking simulations. We found that Phe-343 and Phe-978 showed different conformations depending on the ligand, whereas Phe335, Phe732, and Phe728 adopted almost the same conformation in all simulations. Then, we choose as flexible residues Phe-343, Phe-978, and other amino acids that interact with the ligands according to the docking results with model A.

Our results pointed to Autodock Vina as the best solution. We computed 20 docking runs for each compound using the default parameters for the rest of the variables, and model B as target. This system was also able to reproduce the binding mode of the inhibitor co-crystallized in the mouse experimental structure (ligand named QZ59, PBD code 3G60). A more detailed explanation about the selection and validation of the docking protocol is given in the original research previously published (Palestro et al., 2014). This same procedure has been used here to predict the interaction between CBD and P-gp.

### Quantification and Statistical Analysis

Microscopic images were taken using an Olympus IX-81 microscope equipped with a DP71 camera (Olympus, Japan). Morphometrical and densitometric analyses were performed with ImageJ (NIH) and statistical analysis was done with GraphPad Prism software. Glial morphology was evaluated in a central field of 15 hypoxic and 15 non-hypoxic wells.

The fluorescence intensity was evaluated as a parameter of Rho-123 retention in cell cultures. After checking the normal distribution of the data, differences were analyzed by one-way ANOVA and Student Newman Keuls post-test comparison or by two-tailed Student *t*-test.

## Results

### Effect of CBD on Glial and Endothelial Cells

#### *In vitro* Studies

**FIGURE 2:**
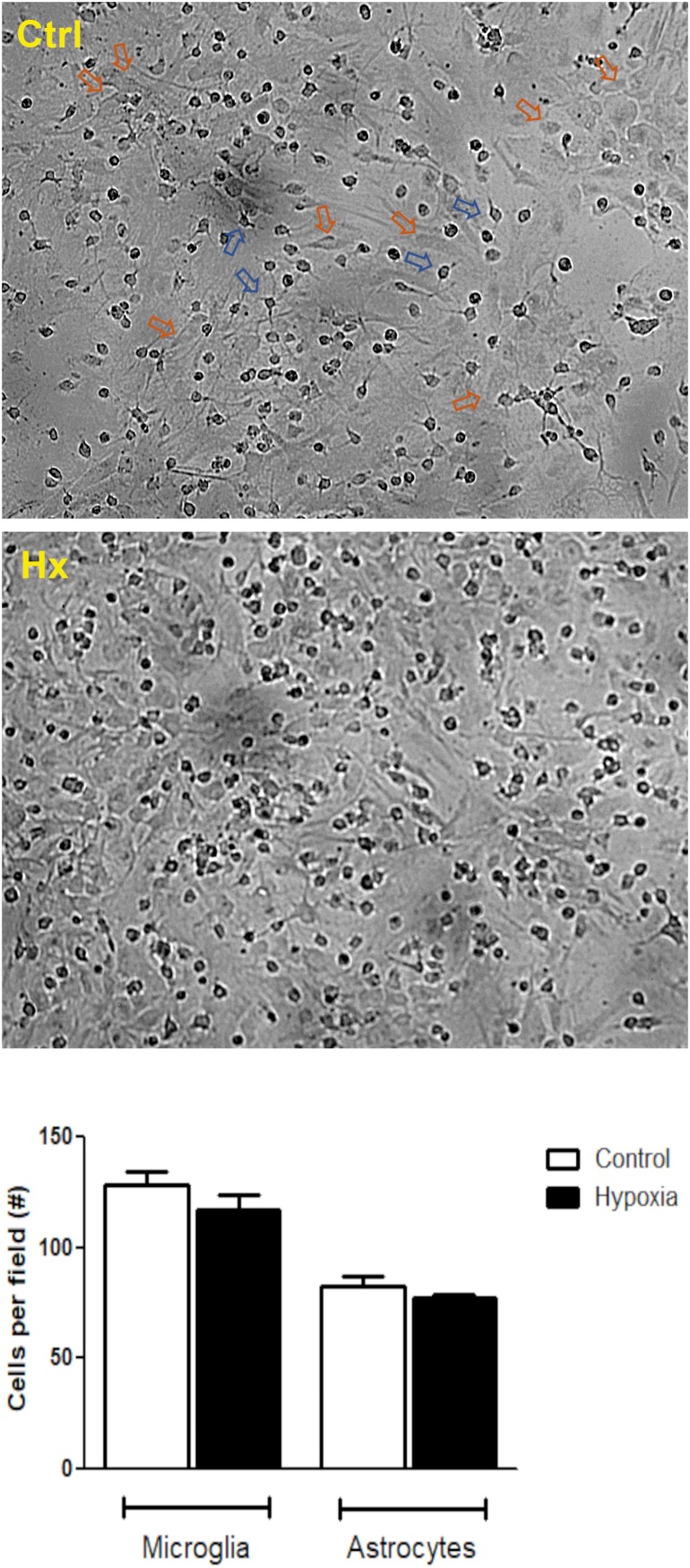
Hypoxia effect on glial mixed culture. Representative phase contrast images showing glial mixed culture. Morphological differences between microglia and astrocytes are indicated by blue or orange arrows, respectively. Glial mixed cultures exposed to control conditions (DMEM, upper image) or chemical CoCl_2_-induced hypoxia (bottom image). Images were acquired at total magnification of 40×.

Initially, we tested the effect of different concentrations of CBD (5, 50, and 100 μM) on glial cells under hypoxia conditions. Microglia and astrocytes were identified based on morphological clues established by Giulian and Baker (1986) and Gebicke-Haerter et al. (1989). While the astrocytes grow at the bottom of the plate with a large soma and long processes, the microglial cells grow on top of them, characterized by a small soma and few prolongations (Figure 2). No changes in the number of glial cells were registered under hypoxia, along the experiments (control: microglia/field = 128.6 ± 6.05 and astrocytes/field = 82.40 ± 4.675; vs hypoxia treatment: microglia/field = 116.8 ± 7.123 and astrocytes/field = 77.00 ± 1.51). Besides, the cell ratio between astrocytes and microglia remained constant during experiments (control = 0.6436 ± 0.031 vs hypoxia = 0.6724 ± 0.054; *p* = 0.2982).

**FIGURE 3:**
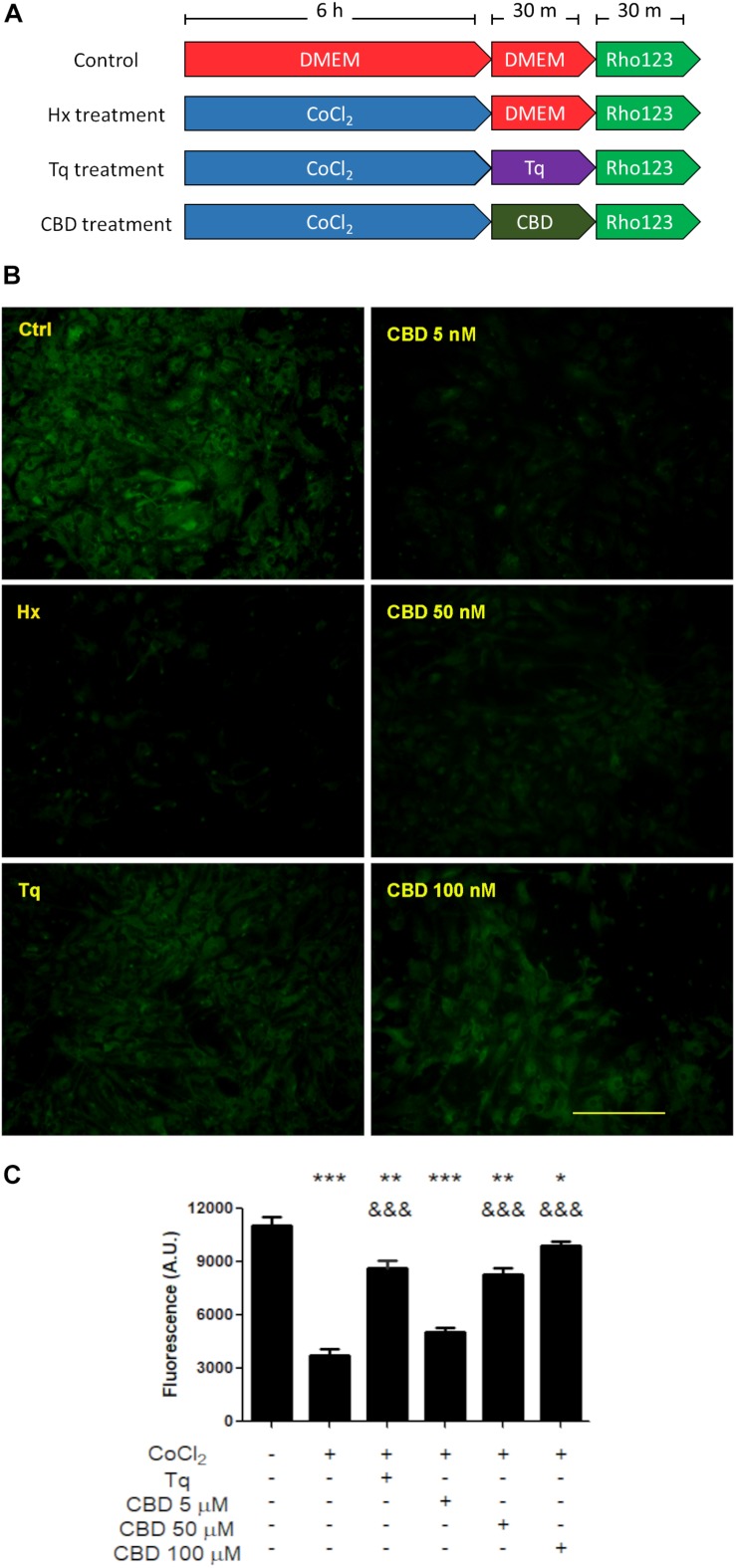
Effect of CBD on glial cells. **(A)** Schematic representation of the treatments and temporal profile applied on mixed glial cell cultures. **(B)** Representative images of the Rho-123 retention by glial culture after each treatment. **(C)** Quantification of the Rho-123 retention as fluorescence intensity for each treatment. At least 20–30 astrocytes were measured in a central field per well, but microglia were not included. Bars represent the mean ± SEM. The differences were analyzed by one-way ANOVA (*p* < 0.001) and Bonferroni post-test. Comparisons with the control were represented by an asterisk (*) while (&) represents comparisons against hypoxia treatment.

On the other hand, when Rho-123 efflux was assayed under hypoxic conditions, astrocytes showed a decreased Rho-123 retention, while in microglia, it remained unchanged. In this context, Tq treatment recovered the Rho-123 retention in astrocytes from mixed glial cultures (Figure 3). Noteworthy, CBD produced a similar retention of Rho-123 to that achieved by Tq. Moreover, astrocytes exposed to hypoxia and subsequent CBD treatment recovered Rho-123 retention in a concentration-dependent manner (5–100 μM) (Figure 3).

**FIGURE 4:**
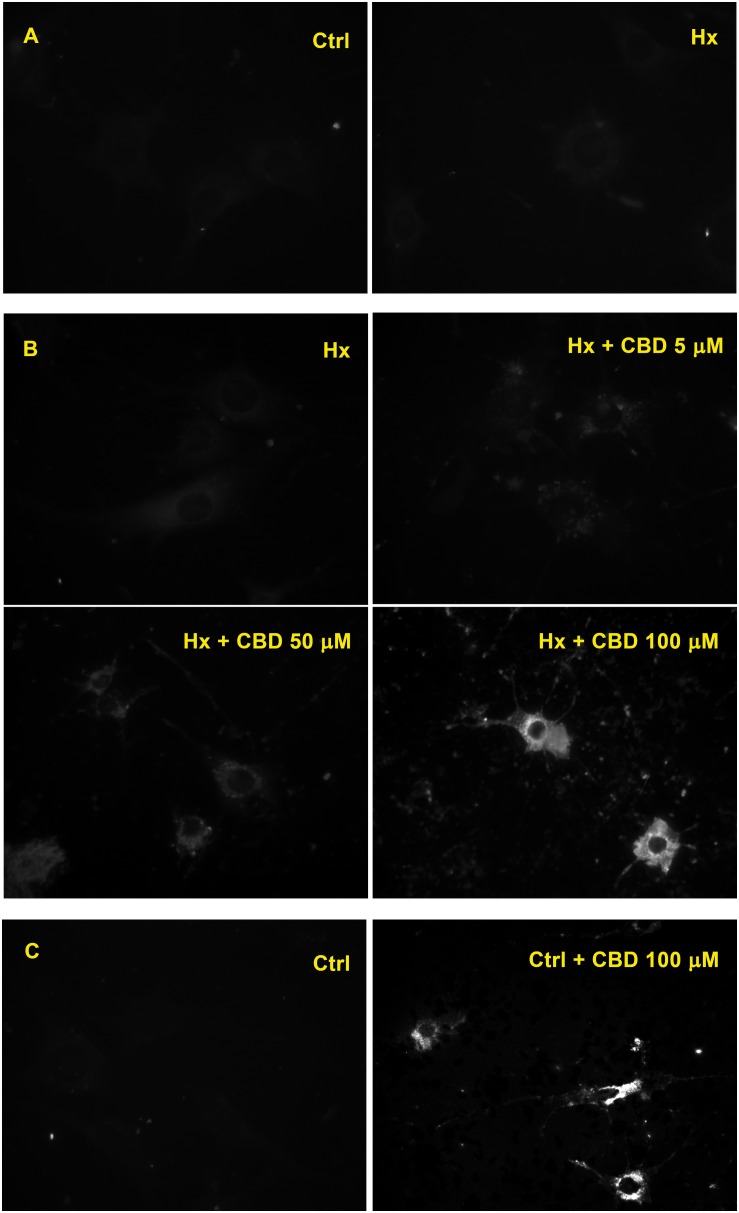
Effect of CBD on endothelial cells. **(A)** Fluorescence microscopy of H5V cells. **(B)** H5V cells exposed to hypoxia in the presence of CBD (5, 50, or 100 μM). **(C)** H5V cells, in normoxia, exposed to CBD (100 μM). All images were obtained with a magnification of 60×.

When Rho-123 retention was evaluated on H5V endothelial cell cultures exposed to hypoxia, a similarly low level of the fluorescent compound retention was observed in both control and hypoxic conditions (Figure 4A). Nevertheless, when H5V cells, under hypoxia, were exposed to CBD 5, 50, or 100 μM, fluorescence was increased in a concentration-dependent manner (Figure 4B). Additionally, when H5V cells, in normoxia, were exposed to CBD 100 μM, Rho-123 retention was also increased (Figure 4C).

#### *In silico* Studies

**FIGURE 5:**
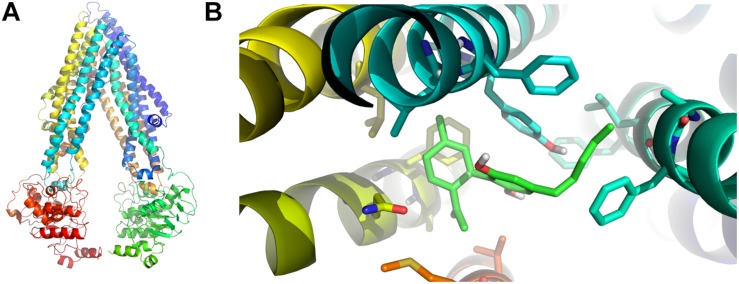
*In silico* binding between P-gp and CBD. **(A)** Homology model of human P-gp. **(B)** Predicted binding mode between CBD (light green) and P-gp amino acid residues in the binding foci. The aromatic residues shown in the figure correspond to Phe 728 and Tyr 307 (both in light blue), the latter of which seems to be involved in the most relevant specific interaction through pi stacking.

Since CBD blocked the active Rho-123 efflux, and this activity is a known property of P-gp, we performed *in silico* studies to test the possible interaction between CBD and P-gp. In our docking experiment, the lowest energy pose displayed an estimated binding energy of -9.0 kcal/mol. These data suggest that CBD may act as a P-gp substrate (thus behaving as a competitive inhibitor). Figure 5 shows the homology model of human P-gp and the predicted binding mode of CBD to the docking binding site.

## Discussion

**FIGURE 6:**
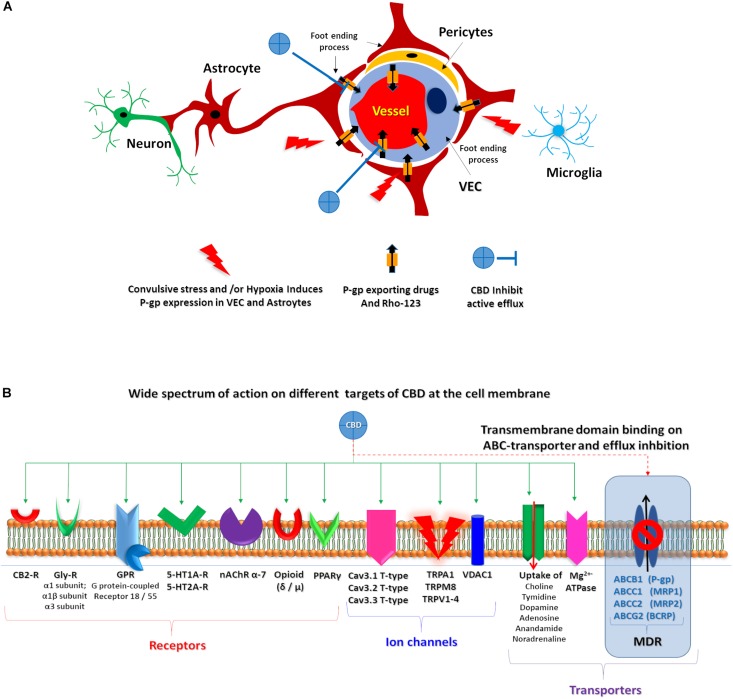
CBD proposed targets. **(A)** Schematic representation of the proposed CBD action on endothelial cells and astrocytes after convulsive stress and or hypoxia. **(B)** Representation of wide spectrum of CBD target on a membrane cell. Green solid arrows identified several described CBD target. Red dashed arrow highlights the proposed CBD action on ABC transporters.

Refractory epilepsies have been associated with ABC transporter overexpression (Löscher and Potschka, 2002; Lazarowski et al., 2007b; Löscher et al., 2011), in direct relation with the recently proposed pharmacokinetic hypothesis (Tang et al., 2017; Leandro et al., 2019). In this scenario, ABC transporter overexpression at the NVU level could be responsible for sub-therapeutic levels of AEDs at the brain parenchyma (Figure 6A).

Our present study demonstrates that CBD inhibits the active efflux of Rho-123, a recognized P-gp substrate, in two prominent members of the NVU such as astrocytes and vascular endothelial cells. Furthermore, these results were more evident under chemically induced hypoxia, a condition in which P-gp is induced to be overexpressed *in vivo*, in vascular endothelial cells, astrocytes, and neurons (Lazarowski et al., 2007a; Merelli et al., 2011a). As shown in Figure 1, CoCl_2_ inhibits the prolyl hydroxylase, a HEM enzyme that is regulated by oxygen concentration. Inhibition of prolyl hydroxylase allows the stabilization of HIF-1α with its concomitant gene expression to hypoxic response. Furthermore, 50 and 100 μM of CBD recovered a similar amount of Rho-123 intracellular retention than the specific P-gp inhibitor Tq, suggesting that these concentrations of CBD produce an inhibitory effect on ABC transporter efflux activity, particularly on P-gp, as demonstrated on trophoblast cell line, mouse embryonic fibroblast, Caco-2, and LLC-PK1/MDR1 cells (Holland et al., 2006; Zhu, 2006; Feinshtein et al., 2013).

Our docking analysis suggests that CBD could act as a competitive inhibitor of P-gp-mediated transport. Also, we observed that CBD inhibited the Rho-123 efflux of non-hypoxic H5V cells, a type of vascular endothelial cells that normally express ABC transporters (Figure 4). These data clearly are in accord with the “transporters hypothesis,” where MDR phenotype is related with P-gp overexpression at this type cell level.

From these observations, several aspects should be considered. Despite the fact that different new AEDs have been developed, some of them involving novel modes of action, 30% of epileptic patients continue to have seizures, being carriers of the so-called MDR epilepsy phenotype. The “transporter hypothesis” suggests that brain overexpression of several ABC transporters is responsible for the MDR phenotype in patients with RE. As previously reported by our group and other authors, brain overexpression of P-gp appears as one of the more common mechanisms related with RE carriers of this MDR phenotype. Several reports have also described the potential effects of P-gp inhibitors as adjuvant therapy next to AEDs, to achieve better control of seizures in different cases of drug-resistant epilepsies (Lazarowski et al., 2007b; Robey et al., 2008).

Interestingly, for several thousand years, humanity has given medicinal use to *Cannabis sativa* (Marijuana), even for the treatment of epileptic patients. In addition to their behavioral and psychotropic effects, cannabinoids have complex pharmacological effects by binding to two specific plasma membrane G protein-coupled receptors: the CB1 receptor expressed mainly in the brain and in some peripheral tissues and the CB2 receptor expressed mainly outside of the CNS have been described (Pertwee and Ross, 2002). Several experimental studies have documented that the endocannabinoid system is strongly activated by seizures, and the upregulation of CB1R activity has antiseizure effects; furthermore, depending on the type of pharmacological action on CB1R, it can both increase and suppress the seizure-like discharges in hippocampus (Marsicano et al., 2003; Deshpande et al., 2007a, b).

The antiepileptic effects of some cannabinoids were revised, showing that they can act on multiple targets (Friedman and Devinsky, 2016). Because CBD lacks THC psychotropic action, it could be an excellent alternative candidate to treat patients with RE (Cilio et al., 2014); in fact, oral CBD has recently been approved as an add-on treatment of severe drug-resistant epilepsies, such as Dravet and Lennox–Gastaut syndromes (Devinsky et al., 2019). However, due to its complex pharmacology, its specific mechanism(s) of action in epilepsy is (are) yet to be described. Whereas the wide spectrum of known CBD targets are not directly related to the MDR phenotype observed in RE (Figure 6), the multi-target nature of CBD could explain its effects on RE, as multi-target drugs are being actively explored as potential treatment of complex disorders, in line with a network pharmacology/systems biology perspective (Margineanu, 2014, 2016).

In previous experimental studies, our group has provided *in vivo* evidence that focal brain injection of CoCl_2_ (1 mM) induces P-gp expression surrounding the lesion site in neurons, astrocytic end-foot, and their related endothelial cells on blood vessels, and that higher CoCl_2_ doses (200 mM) resulted in additional P-gp immunostaining of the whole astrocytic and neuronal soma (Lazarowski et al., 2007a). Nuclear translocation of hypoxia-inducible factor 1-alpha (HIF-1α) was also observed in this experimental paradigm (Caltana et al., 2009) along with erythropoietin receptor (Epo-R) and P-gp co-expression in neurons, astrocytes, and vascular endothelial cells. Interestingly, both Epo-R and P-gp expression are inducible by HIF-1α (Comerford et al., 2002; Semenza, 2009). On the other hand, we also demonstrated that repetitive seizures and/or status epilepticus induce high expression of P-gp in neurons, astrocytes, and vessels, associated with the MDR phenotype (Lazarowski et al., 2004b; Höcht et al., 2007; Auzmendi et al., 2013; Merelli et al., 2019). Furthermore, repetitive seizures and/or status epilepticus also activate HIF-1α and induce P-gp overexpression in heart, which appears to be associated with heart failure and sudden unexpected death in epilepsy (SUDEP) (Auzmendi et al., 2018).

In our current study, a clear inhibition by CBD of Rho-123 efflux was observed in hypoxic astrocytes and vascular endothelial cells. It is known that Rho-123 is a substrate of P-gp; however, Rho-110, a metabolic product of Rho-123, can also be transported by other ABC transporters as MRP2 is also expressed at the BBB (Jäger et al., 1997; Semenza, 2009; Parasrampuria and Mehvar, 2010). According to docking studies presented here, the estimated binding energy of CBD to P-gp suggests that it may act as a weak substrate for the transporter. This observation is in line with the high CBD concentration needed (50–100 μM) to produce a significant retention of Rho-123 compared to a potent TQ specific blocker (5 μM). Previous reports using P-gp knockout mice showed that the lack of P-gp does not limit the brain uptake of CBD in healthy mice (Brzozowska et al., 2016). These reports do not necessarily imply that CBD does not interact with P-gp, especially in the context of high-expression levels of the transporter, as those observed in RE patients (Löscher et al., 2011). Consistent with these evidences, Holland et al. reported that low CBD concentration does not improve uptake of the Rho-123 in CEM/VLB100 cells expressing high levels of P-gp. In contrast, high levels of CBD (10 μM) sensitize such cells to vinblastine, another known P-gp substrate (Holland et al., 2006).

It is known that CBD is a multi-target compound, acting on ionic channels, neurotransmitter receptors, and other transmembrane transporters, with different effects in each of them acting such as activator, modulator, agonist, antagonist, etc. Additionally, not only is CBD metabolized by the enzymatic cytochrome system, but it can also inhibit some of these enzymes, inducing a slowdown in the metabolism of more common AEDs, which is an alternative way of affecting their pharmacokinetics (Rocha et al., 2020). Therefore, CBD effects on ABC transporters are only one among multiple mechanisms through which this drug could have an impact on the MDR phenotype in RE patients.

## Conclusion

Our results indicate that, in addition to the various effects previously described by CBD, this drug can also inhibit the active efflux of Rho-123, a known P-gp substrate, in two types of cells of the NVU, in a similar (though less potent) manner to TQ. Consistently, our *in silico* study indicates that CBD may bind the transmembrane domain of P-gp, possibly acting as a competitive inhibitor. It remains to be studied whether CBD may also impair P-gp-mediated transport in a non-competitive manner. CBD could thus be used as an adjuvant therapy to reverse the MDR phenotype as observed in patients with RE, which could explain its recent approval as an add-on therapy to treat severe refractory childhood epilepsies.

## Data Availability Statement

The datasets generated for this study are available on request to the corresponding author.

## Ethics Statement

The animal study was reviewed and approved by CICUAL.

## Author Contributions

JA, AM, AT, AR, GC, and AL: conceptualization and investigation. JA, PP, AB, and LG: methodology. JA, PP, AB, LG, and AT: software and data curation. JA, PP, LG, AT, GC, and AL: formal analysis. JA, AM, AT, GC, and AL: resources. JA, AM, AT, and AL: writing – original draft preparation, review, and editing. JA and AL: supervision and project administration.

## Conflict of Interest

The authors declare that the research was conducted in the absence of any commercial or financial relationships that could be construed as a potential conflict of interest.
